# Novel Autosomal Recessive Splice-Altering Variant in *PRKD1* Is Associated with Congenital Heart Disease

**DOI:** 10.3390/genes12050612

**Published:** 2021-04-21

**Authors:** Salam Massadeh, Maha Albeladi, Nour Albesher, Fahad Alhabshan, Kapil Dev Kampe, Farah Chaikhouni, Mohamed S. Kabbani, Christian Beetz, Manal Alaamery

**Affiliations:** 1 Developmental Medicine Department, King Abdullah International Medical Research Center, King Saud Bin Abdulaziz University for Health Sciences, King Abdulaziz Medical City, Ministry of National Guard- Health Affairs (MNG-HA), Riyadh 11481, Saudi Arabia; massadehsa@ngha.med.sa (S.M.); albladima@NGHA.MED.SA (M.A.); nalbesher@kacst.edu.sa (N.A.); 2 KACST-BWH Centre of Excellence for Biomedicine, Joint Centers of Excellence Program, King Abdulaziz City for Science and Technology (KACST), Riyadh 11442, Saudi Arabia; 3 Saudi Human Genome Project (SHGP), King Abdulaziz City for Science and Technology (KACST), Satellite Lab at King Abdulaziz Medical City (KAMC), Ministry of National Guard Health Affairs (MNG-HA), Riyadh 11481, Saudi Arabia; 4 Department of Cardiac Sciences, Ministry of the National Guard—Health Affairs, King Abdullah International Medical Research Center, King Saud bin Abdulaziz University for Health Sciences, Riyadh 11481, Saudi Arabia; habshanf@ngha.med.sa (F.A.); chakhounifa@ngha.med.sa (F.C.); kabbanim@ngha.med.sa (M.S.K.); 5 Centogene GmbH, 18055 Rostock, Germany; Kapil.Kampe@centogene.com (K.D.K.); Christian.Beetz@centogene.com (C.B.)

**Keywords:** *PRKD1*, congenital heart disease, splice altering variant, whole exome sequencing, multiple affected

## Abstract

Congenital heart defects (CHDs) are the most common types of birth defects, and global incidence of CHDs is on the rise. Despite the prevalence of CHDs, the genetic determinants of the defects are still in the process of being identified. Herein, we report a consanguineous Saudi family with three CHD affected daughters. We used whole exome sequencing (WES) to investigate the genetic cause of CHDs in the affected daughters. We found that all affected individuals were homozygous for a novel splice-altering variant (NM_001330069.1: c.265-1G>T) of *PRKD1*, which encodes a calcium/calmodulin-dependent protein kinase in the heart. The homozygous variant was found in the affected patients with Pulmonary Stenosis (PS), Truncus Arteriosis (TA), and Atrial Septal Defect (ASD). Based on the family’s pedigree, the variant acts in an autosomal recessive manner, which makes it the second autosomal recessive variant of *PRKD1* to be identified with a link to CHDs, while all other previously described variants act dominantly. Interestingly, the father of the affected daughters was also homozygous for the variant, though he was asymptomatic of CHDs himself. Since both of his sisters had CHDs as well, this raises the possibility that the novel *PRKD1* variant may undergo autosomal recessive inheritance mode with gender limitation. This finding confirms that CHD can be associated with both dominant and recessive mutations of the *PRKD1* gene, and it provides a new insight to genotype–phenotype association between *PRKD1* and CHDs. To our knowledge, this is the first report of this specific *PRKD1* mutation associated with CHDs.

## 1. Introduction

Congenital heart defects (CHDs) are the most common type of birth defects [1], caused by abnormal development of the heart or heart vessels during fetal development. Clinically, CHD manifests in diverse syndromic and non-syndromic phenotypes. There are at least eighteen different recognized CHDs in which the walls, valves, or the blood vessels surrounding the heart are affected, including Ventricular Septal Defect (VSD), Tetralogy of Fallot, Atrial Septal Defect (ASD), Aortic Valve Stenosis (AVS), and Pulmonary Valve Stenosis (PVS) [2]. Globally, CHD prevalence is estimated to range between 19 and 75 cases per 1000 live births and is increasing [3]. CHDs have a multifactorial etiology including both heritable and environmental factors. Genetic causes can be identified in about 20–30% of CHD cases, recognized to be an underestimate [4], while only about 2% of CHDs are attributed to known environmental risk factors such as diabetes during pregnancy [5]. The severity of CHD symptoms and their impact varies according to the nature of the heart defect, with some mild forms, CHD appear with no symptoms [6], while other severe forms require critical surgical interventions, prescribed medications, regular checkups, and monitoring throughout the patients’ lives. These, in turn, have a drastic effect on the patient’s quality of life; moreover, is associated with a substantial global burden [7,8].

The increased incidence of CHDs, in particular pulmonic stenosis, VSD, ASD, and AVSD [9] is prominently high among inbred populations, suggesting that recessive genetic components may play a role. Genomic sequencing approaches have been indispensable to expose the inherited genetic causes of CHDs among multiple related individuals and have significantly contributed to the basic understanding of cardiac morphogenesis and the development of molecular inspired therapeutic and preventative procedures. These approaches have led to the discovery of novel CHD-associated genes, including *NRP1*, *PRDM1*, *ADAMTS19* and, of particular importance to our study, *PRKD1* [10,11,12,13]. So far, only one study revealed the autosomal recessive mode of inheritance in CHDs related to *PRKD1* [11].

In the present study, we report the identification of an autosomal recessive *PRKD1* variant associated with CHDs in three female children of a consanguineous Saudi family based on whole-exome sequencing (WES). The affected individuals exhibited various cardiac anomalies including pulmonic stenosis, tricuspid regurgitation, and truncus arteriosus. WES revealed a novel *PRKD1* splice variant in the affected individuals. To the best of our knowledge, this is the first report of autosomal recessive *PRKD1*-related cardiac phenotypes associated with this particular splice variant. 

## 2. Materials and Methods

### 2.1. Patient Description and Ethical Considerations

**Figure 1:**
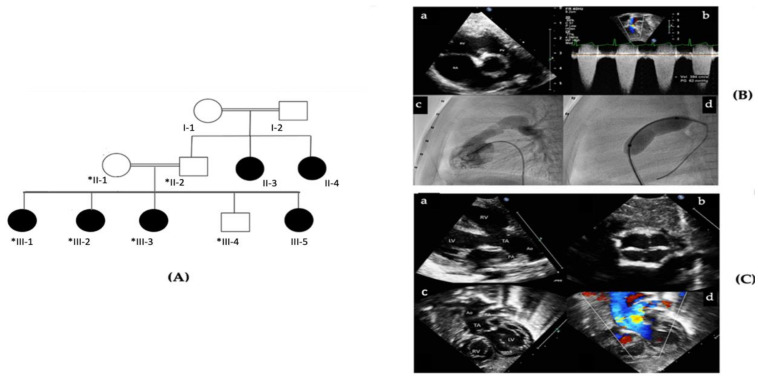
(**A**) Pedigree of a consanguineous family with members affected with congenital heart defects. Pedigree depicts an autosomal recessive mode of inheritance. Double lines are indicative of consanguineous union. Females and males are represented with circles and square symbols, respectively. Clear symbols signify healthy individuals, and filled symbols signify individuals affected with cardiac diseases. +/+ denotes individual with (c.265-1G>T) homozygous mutation in the *PRKD1* gene and +/− denotes carriers for a heterozygous (c.265-1G>T) *PRKD1* variant. The individual numbers labelled with asterisks indicates the samples which are available for the studies. (**B**) Echo images from the proband patient (III-1) with pulmonary stenosis. a: Parasternal short axis view showing thickened and stenotic pulmonary valve. b: Doppler measurement of the flow velocity across the pulmonary valve with maximum peak gradient of 62mmHg. c: Right ventricular angiography showing thickened and stenotic pulmonary valve. d: Balloon dilatation of the pulmonary valve with a waist at the valve area. (**C**) Echo images from patient III-2 with Truncus arteriosus. a: Parasternal long axis view showing a large ventricular septal defect with truncus arteriosus, aorta (Ao) and pulmonary artery (PA). b: Short axis image showing the quadricuspid truncal valve. c, d: Subcostal 2D and colour images showing the truncus arteriosus and its division to aorta (Ao) and pulmonary artery (PA). LV: left ventricle. RV: right ventricle.

The proband patient in this study is a 10-year-old female belonging to a Saudi consanguineous family (III-3, Figure 1A). The family consists of two healthy parents and five children (4 affected females and 1 healthy male). The proband has been diagnosed with pulmonary stenosis and tricuspid regurgitation. Her younger sister (III-2), 9-year-old, suffers from pulmonic stenosis (PS), truncus arteriosus (TA), and ventricular septal defect (VSD). Both siblings were diagnosed and treated at the Department of Pediatric Cardiology, King Abdulaziz Medical City, Ministry of National Guard Health Affairs, Riyadh, Saudi Arabia (Figure 1B,C). The other two affected females (III-3, III-5), 7- and 3-year-old, respectively, showed mild cardiac anomalies that healed on their own. Table 1 summaries the clinical features and medical history of the patients involved in this study. Similarly, the father’s two sisters (II-2 and II-3) suffered from congenital heart anomalies, specifically a septal defect and the condition healed spontaneously without medical intervention in both cases. Ethical approval for this study was obtained from the Institutional Review Board Committee at King Abdullah International Medical Research Centre, Riyadh, and a written consent form was signed by the patients’ parents.

### 2.2. Genomic DNA Extraction

DNA was extracted from blood samples utilizing a QIAamp DNA Micro kit (Hilden, Germany). DNA quantification and quality checking were performed using a NanoDrop™ spectrophotometer following standard procedures.

### 2.3. Whole-Exome Sequencing (WES) 

WES was performed through Centogene Company (Centogene GmbH, Rostock, Germany). DNA samples of six family members, consisting of three affected individuals (III- 1, III-2, and III-3), both parents (II-1, II-2), and unaffected sibling (III-4). DNA was sequenced using an Illumina platform (Illumina, Inc., San Diego, CA, USA). RNA capture baits against approximately 60 Mb of the human exome (covering > 99% of regions in the Consensus Coding Sequence, Gencode databases, and RefSeq) were used to enrich for genomic regions of interest. Genomic DNA was fragmented using the SureSelect Human All Exon V6 kit (Agilent Technologies, Santa Clara, CA, USA). The generated library was then sequenced to obtain an average of 100× depth of coverage, and approximately 97% of the targeted bases were covered more than 10×. 

### 2.4. Variant Annotation and Filtering

Data interpretation and analysis were carried out by Centogene. The analysis was performed using an end-to-end in-house bioinformatics pipeline with applications including base calling and alignment of reads to the GRCh37/hg19 (GRCh37; http://genome.ucsc.edu/ (accessed on 22 February 2019)) genome assembly. The analysis cascade has been previously described [11]. In summary, primary filtering out of low-quality reads and possible artifacts, and the subsequent annotation of variants was applied. All detected variants were screened against all disease-causing variants reported in the Human Mutations Database (www.hgmd.cf.ac.uk/ac/index.php (accessed on 22 February 2019)), the Centogene mutation database (CentoMD) and ClinVar (https://www.ncbi.nlm.nih.gov/clinvar/ (accessed on 22 February 2019)). Evaluation of variants focused on coding exons and the flanking +/−20 intronic bases. All pertinent inheritance patterns and the family history, as well as clinical information, were used to evaluate and eventually identify the relevant variants. Only variations within genes potentially related to the proband’s medical condition were reported.

### 2.5. Sanger Sequencing

The identified *PRKD1* variant (c.265-1G>T) was subjected to Sanger sequencing for all the family members under investigation using standard methods (Centogene GmbH, Rostock, Germany). 

### 2.6. mRNA Analysis

To enable analysis of the *PRKD1* transcript, separate blood samples were collected using the PAXgene^®^ Blood RNA Tube system (BD Biosciences, Franklin Lakes, NJ, USA). The samples were used to prepare mRNA according to the manufacturer’s instructions. Subsequent PCRs were carried out using standard reagents and equipment. We used Primer3Plus software for primer design (http://www.bioinformatics.nl/cgi-bin/primer3plus/primer3plus.cgi (accessed on 22 November 2020)). We applied a forward primer that binds in exon 1 and a reverse primer that binds in exon 3 (Table 2). The size of the expected product was 351 bp. Templates for the PCRs were from the index, from the heterozygous mother and from a wild-type control. 

## 3. Results

### 3.1. Identification of a Splice Site Mutation in PRKD1 

**Figure 2:**
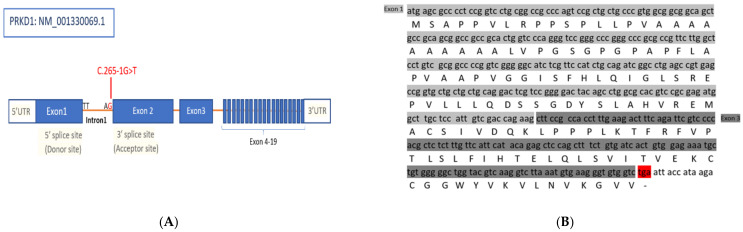
Schematic representation of *PRKD1.* (**A**) structure of the *PRKD1* gene and the splice mutation position (c.265-1G>T) in the variant reported in this study. (**B**) Nucleotide sequence and predicted amino acid sequence alignment of *PRKD1* variant. The exon 2 skipping event detected in *PRKD1* cDNA (NM_0011330069.1: C.265-1 G>T), is predicted to truncate *PRKD1* transcript by the formation of a premature stop codon highlighted in red.

In this study WES was used to investigate the genetic cause behind this clinical presentation. Our analysis revealed a *PRKD1* variant of uncertain significance associated with the disease, which has a single nucleotide substitution (c.265-1G>T) located inintron1 (Figure 2). All three affected females as well as the unaffected father were homozygous for this variant, while the mother and unaffected son were heterozygous (Figure 1). The *PRKD1* variant c.265-1G>T is predicted to disrupt a highly conserved acceptor splice site of exon 2 which would interfere with spliceosome recognition and assembly; and resulting in incorrect splicing. This variant in *PRKD1* has neither been documented in gnomAD nor in HGMD and Clinvar, is, thus, novel. Erroneous splicing events including those caused by mutations in pre-mRNA templates were the main cause of disease in several previously documented cases [14]. We assume that this exon skipping in *PRKD1* transcription may be the cause of the CHDs in this family.

### 3.2. PRKD1 Splicing Analysis

**Figure 3:**
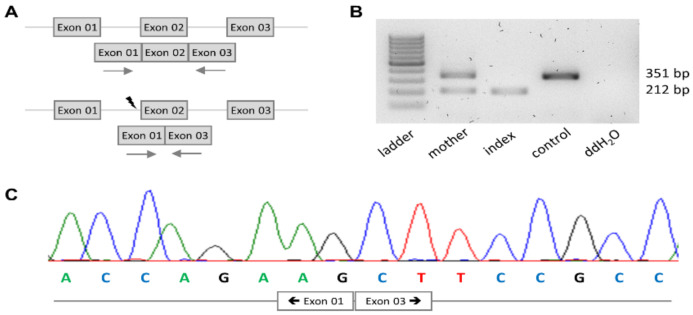
Analysis of the *PRKD1* transcript. (**A**) Strategy to amplify the relevant cDNA region of *PRKD1*, wildtype and mutant scheme on top and bottom, respectively. The flash indicated the position of the variant; arrows indicate PCR primers. (**B**) Results of the reverse transcription PCR. (**C**) Sanger sequence chromatogram of the *PRKD1* transcript in index revealing the absence of exon 2.

The identified variant affected the canonical splice acceptor of exon 2 of *PRKD1*. To determine whether it has an effect on splicing of blood RNA, we applied an exon 2-spanning RT-PCR (Figure 3A). A sample from a wild-type control yielded a single band of the expected size of 351 bp being more intense (Figure 3B). In contrast, a single band of approximately 212 bp was observed when using a sample from the index. In the heterozygous mother, both bands were seen, with the larger one being more intense (Figure 3b). Sequencing of the band from the index revealed exon 1 sequence to be directly followed by exon 3 sequence (Figure 3C). This observation was consistent with skipping of exon 2. The resulting deletion of 139 bp at mRNA level confers a frameshift at protein level and, in turn, generates a premature stop codon in exon 3. A translated protein would thus be severely truncated. Moreover, the mutant *PRKD1* mRNA is predicted to undergo nonsense-mediated mRNA decay [15]. Evidence for the latter to occur is in fact provided by the lower intensity of the smaller mutant band in RT-PCR (Figure 3B). Collectively, these observations render c.265-1G>T a classical loss-of-function variant.

## 4. Discussion

Discovery of the genetic basis of CHDs has been proven to be challenging. Studies have shown that most syndromic CHD can be caused by chromosomal anomalies [16], copy number variations [17], single gene or unknown factors. However, fine mapping of copy number variations (CNVs) in patients with isolated CHD has been used to identify candidate genes that contribute to the origin of pediatric heart disease. The majority of CHD with monogenic inheritance is associated with the known—syndromic presentation. Furthermore, most of them occur sporadically, and families show a clear monogenic mode of inheritance of non-syndromic CHD and cardiac anomalies have been found to result from changes in single or multiple genes with either dominant or recessive mode of inheritance [18]. Phenotypically, both single and multiple genes defects can be translated into syndromic or non-syndromic CHDs. The majority of CHD patients exhibit an isolated non-syndromic CHD phenotype despite this fact, the genetic mechanisms underlying isolated CHD cases remain incompletely understood [4]. Evidence for the genetic basis of isolated CHD comes from familial clustering of cases [19]. In the last two decades, research has primarily focused on gene discovery in non-syndromic forms of CHD, and the list of gene mutations that result in isolated CHDs is rapidly expanding, yet the evidence is variable for each gene [20]. The first genes linked to non-syndromic CHD were the transcription factors including NKX2.5 and GATA4 [18]. Since the involvement of those genes was discovered, a large number of other genes have also been implicated in isolated CHDs, including the transcription factors (TBX1, TBX2, TBX3, TBX5, and MEF2) were observed [21,22] as well as several genes that function in crucial cell signaling pathways like ZIC3, NOTCH1 [23] BMP-2, BMP-4, and TGF- β, and SOX17 [24,25,26].

Here, we present a comprehensive report of clinical and molecular characterization of a consanguineous family of Saudi origin, demonstrating autosomal recessive inheritance of the non-syndromic form of CHD. The affected members showed multiple cardiac phenotypes including pulmonary stenosis, truncus arteriosus and septal defects. By analyzing the WES data, we identified a rare homozygous variant in the *PRKD1* gene in the affected members of the family. The *PRKD1* gene is mapped to Chr14q1. This gene encodes a protein kinase called PKD1. In addition, PKD2 and PKD3 are two related proteins. They are encoded by *PRKD2* and *PRKD3* genes located on 19q13.32 and 2p22.2, respectively. The three proteins have different subcellular localizations and tissue expression patterns and thus carry out independent and diverse functions [27,28]. PKD1 is the major PKD protein in the heart [28]. Upon activation, PKD1 phosphorylates class II histone deacetylases (HDACs), resulting in their disassociation from the myocyte enhancer factor-2 (MEF2) transcription factor. This PKD1 induced disassociation relieves MEF2 repression and thus induces the activation of its targeted genes [24]. MEF2 is expressed in cardiogenic precursor cells and in differentiated cardiomyocytes and is considered as an essential regulator of cardiac myogenesis and the right ventricular development [27]. Unfortunately, the heart phenotype of prkd1−/− is uncharacterized in mice because the *PRKD1* complete knockout caused embryonic lethality [28]. However, Mef2c−/− mice models were found to have abnormalities in heart tube looping, cushion formation and complex vascular malformations [29,30,31,32], suggesting that the protein truncating *PRKD1* variant may be responsible for the CHD phenotype through inability to relieve MEF2 repression.

In addition, there is increasing evidence that PKD-mediated signaling pathway plays an important role in the cardiovascular system, particularly in the regulation of myocardial contraction, hypertrophy, and pathological cardiac remodeling. Moreover, several gain- and loss- of-function mutations in *PRKD1* have been reported to interfere with normal cardiac morphogenesis [11,31]. It has been shown that *PRKD1* is a potential regulatory factor involved in cardiac neural crest cells (NCCc) migration and patterning at the developing arches of the heart [33]. Therefore, loss-of-function mutations in the *PRKD1* gene is predicted to contribute to the pathophysiology of congenital cardiac defects such as TA and Aberrant origin of the right subclavian artery. This is consistent with the TA phenotype identified in our patients with the truncating PRKD1 mutation. We believe that *PRKD1* is the most compelling candidate for TA pathogenesis in our patients [34].

Some *PRKD1* variants have been linked to various heart defects, such as truncus arteriosus, conotruncal heart defects and many other cardiac anomalies as listed in Table 3. Sifrim et al. reported that missense mutations in the *PRKD1* gene were significantly enriched in patients with congenital heart defects and ectodermal dysplasia (CHDED) [33]. They identified de novo missense mutations in *PRKD1* in three syndromic CHD cases. These mutations included an amino acid substitution (p.Le299Trp) within the CysII rich regulatory domain of *PKD1* and a (p.Gly592Arg) mutation within the *PKD1* catalytic domain. Both mutations suggested a dominant gain of function in *PRKD1*.Thus, an autosomal dominant *PRKD1*-associated OMIM phenotype termed congenital heart defects, and ectodermal dysplasia was recognized (OMIM: 617364). Jin et al. also reported few dominant loss-of-function heterozygous mutations in *PRKD1*, in one patient with heterotaxy syndrome and in two patients with conotruncal heart defect (CTD) [10]. 

The pedigree of the affected family suggests that this novel *PRKD1* variant is autosomal recessive. Both parents were unaffected, while three out of four of their children had CHDs. All affected daughters were homozygous for the variant, while their unaffected brother was heterozygous (Figure 1). Although several autosomal dominant *PRKD1* mutations associated with CHDs have been reported [8,16], only one autosomal recessive CHD-associated *PRKD1* variant has previously been identified in Saudi patients [11], this finding suggests that recessive variants in the dominant gene *PRKD1* should not be ruled out before careful consideration. Despite the prevalence of autosomal dominant *PRKD1*-associated phenotypes, recessive forms were not previously observed. Monies et al. (2017) identified recessive variants for 11 different genes for which only dominant diseases had been reported [35]. 

Interestingly, homozygosity for the *PRKD1* variant (NM_001330069.1: c.265-1G>T) reported here was also detected in the unaffected father. It is possible he might have had a mild CHD phenotype which was cleared at an early age. However, it is also possible that the *PRKD1* variant is gender-limited, as only female family members, including the three daughters and the father’s two sisters, were diagnosed with CHDs. Notably, Shaheen at al. also have reported a recessive PRKD1 variant in two female patients [11]. This would not be the first time that sex limitation was considered as a possibility for the genetic determinants of heart disease. Tsagaris et al. posited that cardiac disease in a large family was probably caused by a sex-limited autosomal dominant gene [36]. Additionally, Monteleone and Fagan discovered CHDs in all males in three generations of a family and hypothesized that it was either due to X-linked recessive inheritance or autosomal dominant inheritance with sex limitation [36]. It is unclear whether the novel *PRKD1* variant is truly sex-limited, but it is remarkable that all affected individuals were females and though the father was homozygous for the variant, he showed no CHD phenotype. More research is needed to determine what role, if any, sex plays in the described novel autosomal recessive *PRKD1* variant-associated CHD phenotypes.

The *PRKD1* variant reported in this study, identifies the genetic cause of non-syndromic CHD in a family of multiple affected children. Thus, we report a novel autosomal recessive *PRKD1* variant linked to CHDs. The detection of a homozygous variant in this gene indicates a possible genetic diagnosis of *PRKD1*-associated cardiac phenotypes with an autosomal recessive mode of inheritance.

## 5. Conclusions

Taken altogether, the present study of a Saudi consanguineous family with isolated CHD supports a previous study arguing that biallelic truncating mutation in the *PRKD1* gene may cause non-syndromic CHD. Our finding expands the mutation spectrum of the *PRKD1* gene and demonstrates the substantial impact of consanguineous populations in revealing monogenic forms of complex disorders.

## Figures and Tables

**Table 1:** Clinical features of family members with the (c.265-1G>T) *PRKD1* variant.

	Patient II-3, II-4	Patient III-1	Patient III-2	Patient III-3
Age	NA	10	9	6
Gender	Females	Female	Female	Female
Weight	NA	40	29.6	NA
Height	NA	148	138.5	NA
Cardiovascular Symptoms	NA	Asymptomatic	Respiratory distress	Asymptomatic
Clinical Diagnosis	Septal Defects (not specified)	Pulmonary stenosis	TA type II and VSD	ASD
Mental Development	Normal	Normal	Normal	Normal
Neurological Abnormalities	Normal	Normal	Normal	Normal
Motor development	Normal	Normal	Normal	Normal

Abbreviations; NA: not available, TA: Truncus arteriosus VSD: Ventricle septal defect, ASD: Atrial septal defect.

**Table 2:** Primer sequences for detecting the consequences of the PRKD1 variant (c.265-1G>T) at mRNA level.

Primer	Sequence
Forward	GCATCTCGTTCCATCTGCAG (Exon01)
Reverse	CTCCACAGTGATCACAGAAAGC (Exon03)

**Table 3:** Summary of the PRKD1 variants reported in the CHD Literature.

*PRKD1* Variants	Mutation	Inheritance	CHD Classification	Gender of Human Subjects	Clinical Diagnosis	Ref.
NM_001330069.1: c.265-1G>T	Disruption of acceptor splice side of Exon 2 (Exon skipping event lead to premature stop codon)—(LOF)	Recessive	Non-syndromic CHD	Female	PS, Tricuspid regurgitation	This Study
				Female	TA, VSD	
				Female	ASD	
			NA	Male	Unaffected	
			NA	Male/Female	Healthy control	
NM_002742.2: c.1852 C>T	Homozygous Truncating Mutations in *PRKD1*(LOF)	Recessive	Non-syndromic CHD	Two Females	Truncus arteriosus	Shaheen et al., 2015 [11]
NM_002742.2: c.1774 G>A	De novo missense mutations p. Gly592Arg (Gain of function mutation)	Dominant	Syndromic CHD	Twins Males	Pulmonary valvar abnormality. Coupled with hypoglycemia, jaundice and hypothermia, Delayed speech and language development, Microcephaly, Bilateral conductive hearing impairment, Ectodermal dysplasia, Lipson syndrome.	Sifrim et al., 2016 [33]
					AVSD, Hypotonia, Scoliosis,	
NM_002742.2: c.896 T>G	De novo missense mutations p. leu299Trp (Gain of function mutation)	Dominant	Syndromic CHD	Male	Attention deficit hyperactivity disorder, Microcephaly, Arnold-Chiari type I, Microcephaly, Nystagmus	
Chr14: 30,108,080 G>A	Premature stop codon (p.R243X)	Dominant	NR		Hetrotaxy syndrome, Ebstein anomaly, L-loop corrected transposition	Jin et al., 2017 [10]
Chr14: 30,066,751 G>A	Premature stop codon (p.Q794X)	Dominant	NR		ASD, secundum, patent ductus arteriosus, pulmonary stenosis, valvar	
Chr14: 30,046,444 T>A	Stop-less mutation (p.X913C)	Dominant	NR		Aberrant right subclavian artery, abnormal branching left aortic arch, Aortic stenosis, Bicommissural aortic valve, DORV, LSVC, SDS, tubular hypoplasia of aorta, VSD,	
Chr14: 30,100,011 G>A	Premature Stop codon (p.Q537X)	Dominant	NR		Healthy Control	
ENSG00000184304 G>T	De-novo Missense Mutation	Dominant			ASD, secundum, pulmonary stenosis, Tricuspid stenosis | VSD	

## Data Availability

The data presented in this study are available in this article.

## Abbreviations

LOF	loss-of-function
NA	not applicable
NR	not reported
VSD	Ventricle septal defect
ASD	Atrial septal defect
AVSD	Atrioventricular septal defect
DORV	Double outlet right ventricle
LSVC	Left superior vena cava
SDS	Sudden death syndrome
